# Enzymatic Valorization of Sour Cherry Pomace as Multifunctional Biopolymer Matrix for Active and Intelligent Food Packaging

**DOI:** 10.3390/molecules31213882

**Published:** 2026-10-28

**Authors:** Tatjana Đorđević, Ljiljana Popović, Nevena Hromiš, Senka Popović, Zorica Stojanović, Jelena Vulić, Di Zhang, Jaroslav Katona

**Affiliations:** 1 Faculty of Technology Novi Sad, University of Novi Sad, Boulevard cara Lazara 1, 21000 Novi Sad, Serbia; ljiljana04@tf.uns.ac.rs (L.P.); nevena.krkic@uns.ac.rs (N.H.); madjarev@uns.ac.rs (S.P.); zorica.stojanovic@uns.ac.rs (Z.S.); jvulic@uns.ac.rs (J.V.);; 2 School of Food and Biological Engineering, Jiangsu University, Zhenjiang 212013, China; d.zhang@ujs.edu.cn

**Keywords:** sour cherry pomace, biopolymer films, active packaging, pH sensitivity, enzymatic pretreatment

## Abstract

The increasing generation of food processing by-products has created a need for sustainable strategies for their valorization into high-value materials. The chemical characterization of sour cherry pomace indicates that it is a valuable source of carbohydrates (87.35%) and bioactive and pigment components, containing 21.25 mg GAE/g DW of total phenolics and 1.28 mg Cy3Glu/g DW of total anthocyanins. Therefore, sour cherry pomace was investigated as a single-source raw material for the development of multifunctional biopolymer films with potential applications in active and intelligent food packaging. To modify the polysaccharide matrix and enhance the availability of endogenous phenolic compounds, the pomace was subjected to enzymatic treatment. The treated materials were then processed directly into films without the addition of external phenolic compounds or pigments and compared with films prepared from untreated pomace. The FTIR analysis of the films indicated a higher relative contribution of aromatic components after enzymatic treatment, with the highest antioxidant activities and pH sensitivity observed in the film obtained from pomace treated with enzyme mixture. These findings demonstrate the potential of enzymatically valorized sour cherry pomace as a sustainable and efficient platform for developing intrinsically functional packaging materials, supporting the circular bioeconomy concept, and advancing next-generation food packaging systems.

## 1. Introduction

The rapid and continuous expansion of the food industry, driven by increasing global demand, has resulted in the generation of substantial quantities of by-products. These by-products pose both environmental and economic challenges. However, these materials are rich sources of high-value compounds, particularly biopolymers such as pectin, cellulose, proteins, and polyesters [1,2]. In the context of sustainability and circular economy principles, valorizing food by-products into high-value materials presents a promising strategy for reducing waste while generating added economic value. Bio-based materials have attracted considerable attention due to their potential edibility, biodegradability, and functional and bioactive properties. Compared to conventional petroleum-based packaging materials, bio-based films with active functionalities offer enhanced protection against environmental factors and microbial spoilage, thereby extending shelf life, preserving product quality, and increasing commercial value [3].

Among bio-based materials, polysaccharide-based films, particularly those derived from pectin, cellulose, and chitosan, are the most extensively studied due to their non-toxicity, renewability, and biodegradability. However, these films often have limitations such as low mechanical strength, high brittleness, and limited inherent antioxidant activity, which restrict their practical application. To overcome these drawbacks, various strategies have been employed, including physical blending, chemical crosslinking, lamination, and chemical modification [4,5,6]. To impart active packaging functionality, polysaccharide films are frequently developed as composite systems through the incorporation of polyphenols, which significantly enhance antioxidant and antimicrobial properties [7,8]. Natural phenolic compounds are particularly attractive due to their safety, abundance, and multifunctional bioactivity, including antioxidant and antimicrobial effects [9]. Additionally, the incorporation of natural pigments enables the development of intelligent packaging systems capable of real-time monitoring of food freshness through visible color changes. Anthocyanins, water-soluble pigments with inherent antioxidant activity and pH sensitivity, are especially promising for such applications [10,11]. Their structural transformations under varying pH conditions result in distinct color changes, making them suitable for pH-responsive packaging systems [12,13]. Sour cherry pomace, composed primarily of skins and pulp, is a rich source of phenolic acids, flavonols, and anthocyanins. These compounds are mainly concentrated in the skin and may account for more than 70% of the total phenolic content [14].

Zhang et al. [15] reported that the presence of endogenous phenolic compounds, which are naturally associated with plant polysaccharides, can promote more favorable interactions within the polymer network and improve structural stability compared with the addition of exogenous phenolic compounds. In this regard, sour cherry pomace is a particularly valuable raw material, as it is rich in both polysaccharides and phenolic compounds, including anthocyanins. The natural coexistence of polysaccharides and phenolic compounds in sour cherry pomace enables the formation of bioactive film matrices without the need for additional functional additives. This offers a unique opportunity for the direct development of polysaccharide–phenolic film networks with consistent physicochemical and functional properties.

Therefore, the utilization of sour cherry pomace represents a sustainable approach to developing biopolymer matrices for the production of both bioactive and intelligent films. To enhance the availability of phenolic compounds and anthocyanins for film formation, enzymatic hydrolysis can be applied to partially release these compounds from the polysaccharide matrix. Such treatment allows for controlled modification of the pomace structure without disrupting the inherent interactions between polysaccharides and phenolics, while potentially enhancing antioxidant activity. Enzymatic extraction technologies offer significant advantages due to their specificity and efficiency, relying on targeted degradation of cell wall components through enzyme-catalyzed reactions or complex enzyme systems derived from microbial metabolism.

Despite the growing body of research on bio-based packaging materials, the direct utilization of sour cherry pomace as an integrated source of polysaccharides and endogenous phenolic compounds for the development of intrinsically active and intelligent films remains unexplored. To the best of our knowledge, no previous study has reported the production of film-forming matrices derived solely from sour cherry pomace that simultaneously exhibit bioactivity and pH responsiveness without the incorporation of external functional additives. In this context, the present study introduces a novel approach based on the enzymatic valorization of sour cherry pomace using cellulase, pectinase, hemicellulase, and their combination, enabling targeted structural modification and enhanced availability of bound phenolic constituents. Enzymatically treated and untreated sour cherry pomace samples were used for mild alkaline extraction and film casting. The obtained films were characterized in terms of their chemical composition, physical properties, and structural features. Their antioxidant activity and pH-responsive behavior were also evaluated to assess their potential for use in active and intelligent food packaging applications.

## 2. Results and Discussion

### 2.1. Chemical Characterization of Sour Cherry Pomace

The proximate composition of sour cherry pomace was determined, and the results are presented in Table 1. The moisture content (10.43%) was higher than that reported by Jūrienė et al. [16]. The protein content (8.4%) was comparable to previously reported values, whereas the fat content was notably lower (0.93%). In contrast to earlier findings, where lipid content ranged from 5% to 12% [16], substantially lower levels were observed in the present study. Carbohydrates were identified as the dominant component, accounting for 87.35%, which is slightly higher than the values previously reported in the literature [16]. Although some compositional parameters were consistent with earlier reports, others showed noticeable deviations. Such variability is expected, as the pomace composition is strongly influenced by factors such as fruit variety, growing conditions, soil characteristics, and the analytical methods used [16]. The total polyphenolic and anthocyanin contents were 21.25 mg GAE/g DW and 1.28 mg Cy3Glu/g DW, respectively, exceeding values previously reported for sour cherry pomace [17] obtained using ethanol aqueous extraction under different conditions for phenolics (14.23 mg GAE/g DW) and anthocyanins (0.41 mg/g DW). In the study by Frusciante et al. [14], phenolic content in hydroethanolic cherry pomace extract was 14.6 mg GAE/g DW, while in the study using pulsed electric fields and ultrasound-assisted extraction, it was 41.60 mg GAE/g DW [18]. Simsek et al. [19] found 14.14 mg GAE/g and 13.78 mg GAE/g of sour cherry pomace with microwave-assisted and conventional extraction, respectively.

The phenolic profile of sour cherry pomace was assessed by ultrasound-assisted extraction using a methanol–water mixture, enabling the recovery of a readily extractable fraction of phenolic compounds. The analysis revealed the presence of several phenolic acids, including caffeic, p-coumaric, sinapic, gallic and ellagic acids, as well as flavonoids like rutin. In the obtained cherry pomace extract, sinapic acid was the most abundant (36.34 mg/100 g DW), followed by coumaric and caffeic acid.

However, phenolic acids in plant-derived matrices are predominantly present in bound forms, typically covalently linked to cell wall polysaccharides, particularly pectin and hemicellulose, through ester linkages [20]. Consequently, conventional solvent extraction methods primarily recover the free and weakly bound phenolic fraction, while a substantial portion remains associated with the structural components of the cell wall [21]. This suggests that additional treatments, such as enzymatic or alkaline hydrolysis, may be required to release bound phenolics and enhance the functional potential of the film-forming solution.

### 2.2. FTIR Spectra of Sour Cherry Pomace

**Figure 1:**
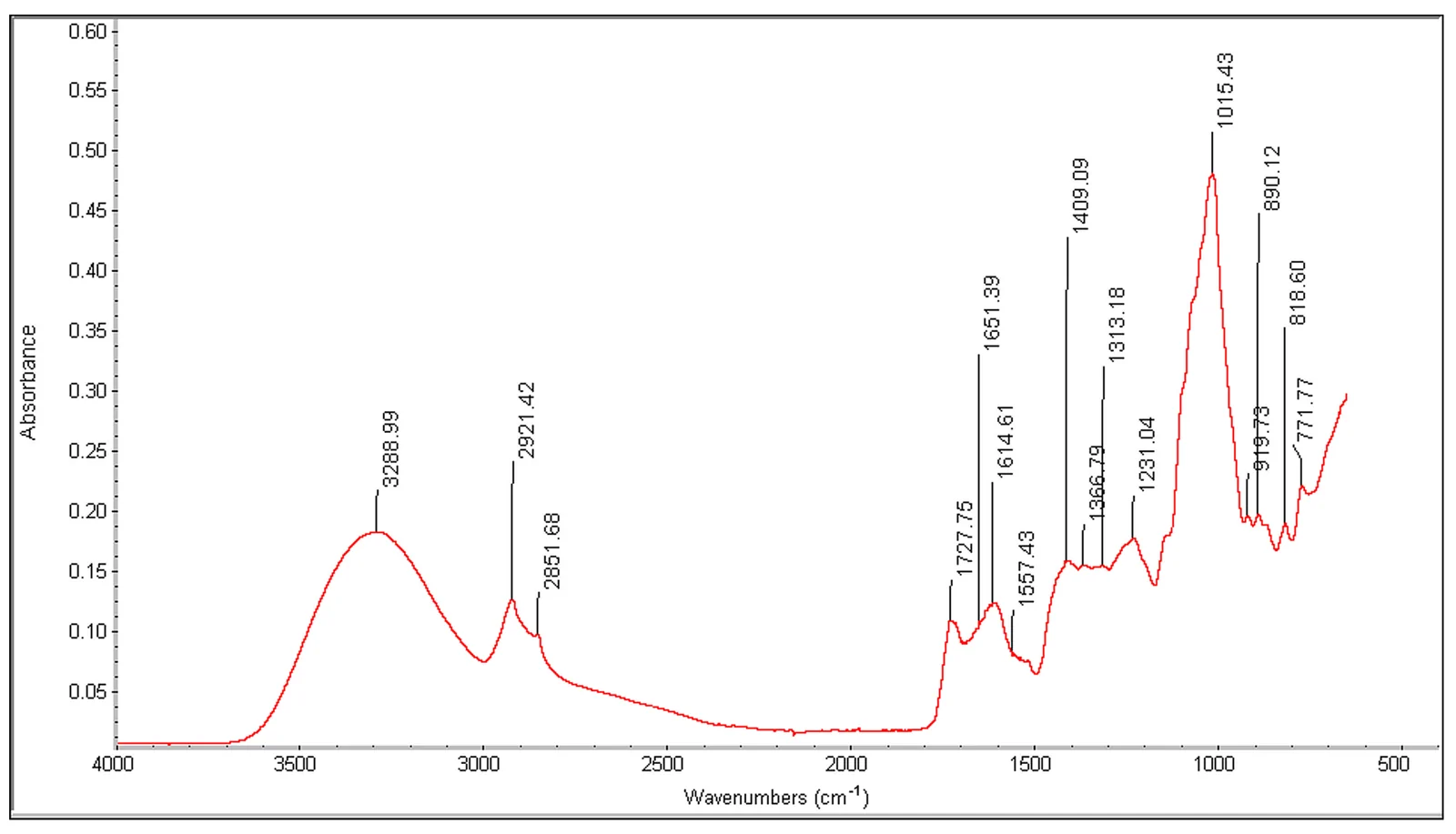
FTIR spectra of untreated sour cherry pomace.

In the FTIR spectra of sour cherry pomace (Figure 1), the dominant peaks at 3288 cm^−1^, 1015 cm^−1^, and 890 cm^−1^ probably originate mainly from the polysaccharides present (cellulose, hemicellulose, and pectin). The peak at 890 cm^−1^ is most probably related to the β-(1→4)-glycosidic bonds characteristic for cellulose, with a possible contribution from hemicelluloses. The presence of ester groups is confirmed by a signal at 1727 cm^−1^, which can be attributed to the C=O vibrations of esterified pectin groups, with contributions from acetylated hemicelluloses, as well as cutin and waxy components present in the untreated material. The peaks at 1614 cm^−1^ and 1409 cm^−1^ can be related to the asymmetric and symmetric vibrations of the carboxylate groups of pectin, but also to the aromatic structures of lignin, indicating the overlap of contributions from different matrix components. The peaks at 1510 cm^−1^ (very small), 1231 cm^−1^, as well as at 818 cm^−1^ and 771 cm^−1^, are most likely related to the aromatic structures of lignin and polyphenolic compounds. Intense peaks at 2921 cm^−1^, with a partial contribution in the region of 1728 cm^−1^, indicate the presence of aliphatic or lipid fractions (waxes, cutin). The presence of proteins can be associated with the identified peak at 1557 cm^−1^ (most likely the amide II band), while amide I and III are overlapped with polysaccharide and lignin signals in the ~1651 cm^−1^ band and 1330–1220 cm^−1^ region [22,23,24,25,26].

### 2.3. Enzymatic Treatment of Sour Cherry Pomace

**Figure 2:**
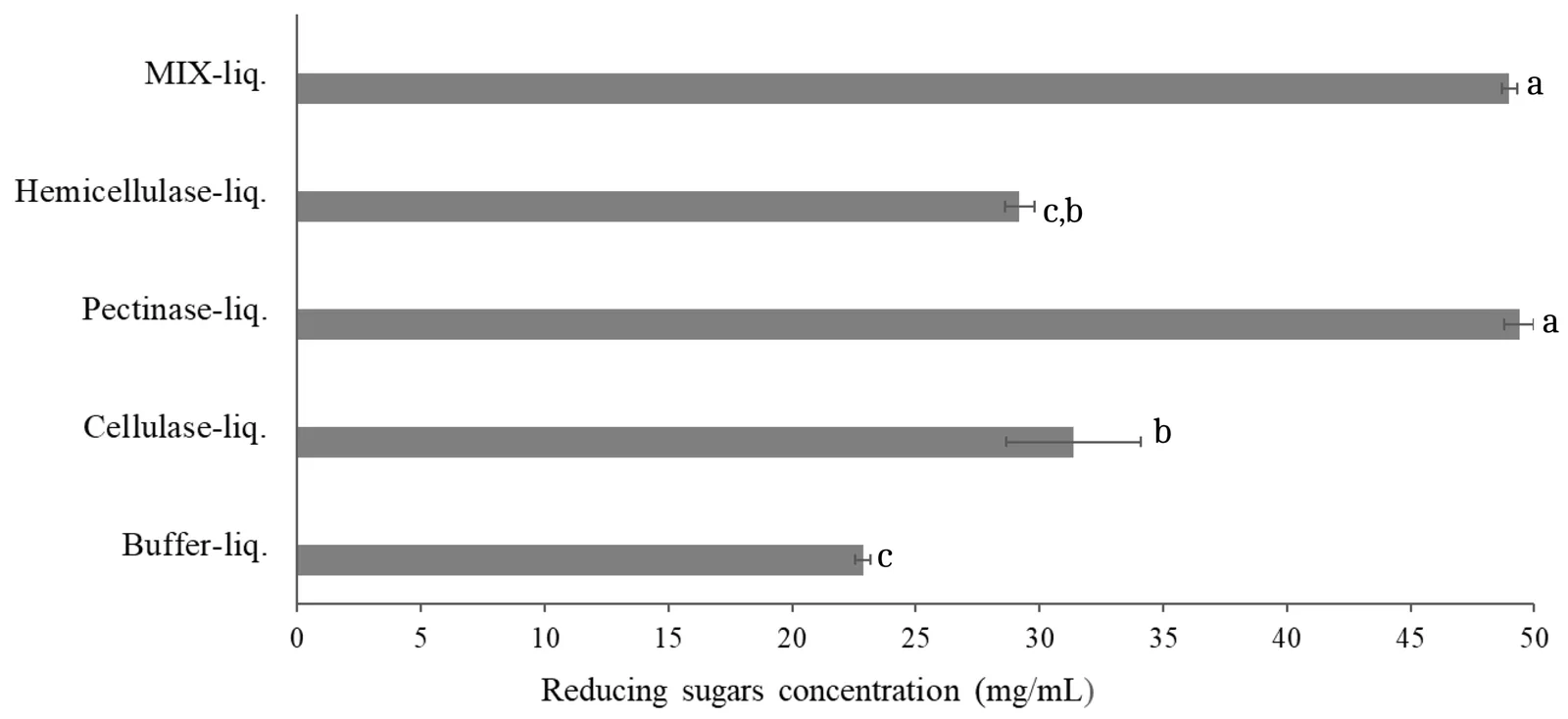
Reducing sugar release in liquid fractions during treatment of sour cherry pomace. Different letters represent the significant differences according to Tukey’s HSD test (*p* < 0.05).

As discussed above, many phenolic compounds are bound to the polysaccharide components of the cell wall, which limits their accessibility. To improve their release, cellulase, pectinase, and hemicellulase were used as enzymatic pretreatments to partially break down the cell wall structure of the pomace matrix. These hydrolytic enzymes were used for pretreatment to partially degrade structural polysaccharides in the pomace matrix. The dried pomace samples treated with buffer alone, cellulase, pectinase, hemicellulase, or the enzyme mixture were designated buffer, cellulase, pectinase, hemicellulase and MIX. The corresponding liquid fractions were assigned buffer-liq., cellulase-liq., pectinase-liq., hemicellulase-liq., and MIX-liq. The enzymatic hydrolysis was monitored by measuring the release of reducing sugars in the liquid fractions, and the results are presented in Figure 2. The results revealed that the release of reducing sugars depended on both the type of enzyme and whether they were applied individually or in combination. As shown in Figure 2, sour cherry pomace treated with pectinase and an enzyme mixture resulted in the highest release of reducing sugars, indicating the most effective disruption of the pomace structure. Among the individual treatments, pectinase exhibited the highest efficiency, whereas hemicellulase was the least effective compared to cellulase and pectinase. These findings are consistent with previous reports on grape pomace, which showed that hemicellulase has lower hydrolytic efficiency than pectinase and cellulase during enzymatic treatment [27]. Because cellulase, pectinase, and hemicellulase act on distinct polysaccharide components and linkages within the plant cell wall [27], combining their activities could enable more effective cell wall deconstruction, promoting the release and recovery of bioactive compounds and potentially enhancing the functional properties of the resulting biopolymer films.

### 2.4. Chemical Characterization of Buffer and Enzyme-Treated Sour Cherry Pomace

The proximate composition of buffer and enzyme-treated sour cherry pomace samples is summarized in Table 2. Differences among samples are indicated by distinct letters (Tukey’s HSD, *p* < 0.05). Moisture content ranged from 2.87 in the hemicellulase-treated sample to 6.46% in the pectinase-treated sample, reflecting variations in water retention associated with the applied treatments. Ash content remained relatively constant across samples (5.59–6.21%), suggesting that mineral composition was not substantially affected by enzymatic treatment. Protein content showed no significant differences among treatments (*p* > 0.05), ranging from 10.67 to 12.47%. Fat content was low overall, as expected for cherry-derived materials (1.44–2.01%), with slightly higher values observed in pectinase- and MIX-treated samples. Carbohydrates, calculated by difference, represented the predominant fraction (79.72–82.02%). As these values were obtained by subtraction, they should be interpreted as a residual fraction that may also include other non-quantified constituents.

### 2.5. Dietary Fiber Content in Buffer and Enzyme-Treated Sour Cherry Pomace

The fiber content in buffer and enzyme-treated sour cherry pomace is presented in Table 3. All enzymatic treatments resulted in a reduction in IDF content compared with the buffer-treated sour cherry pomace, with statistically significant differences observed following hemicellulase, cellulase, and MIX treatments. The greatest reduction was observed in the cellulase-treated sample. This reduction may be attributed to the partial hydrolysis of the cellulose fraction, resulting in structural disintegration, which is consistent with previous findings [28]. The soluble dietary fiber content varied depending on the enzyme applied. The highest SDF content was observed after the action of hemicellulase, whereas the lowest was detected in the pectinase-treated pomace. This decrease may be explained by the degradation of the pectin structure, supported by the overall reduction in carbohydrate content observed when pectinase was used individually. Nevertheless, it can be concluded that the applied hydrolytic enzymes appear to induce structural reorganization of the pomace matrix, modifying the SDF and IDF balance without extensive degradation. These findings are consistent with the proximate composition data of buffer and enzyme-treated sour cherry pomace (Table 2).

### 2.6. Appearance Characteristics of Film

Based on the obtained and presented results, CEL and MIX treatments were selected for subsequent film preparation. The aim of enzymatic pretreatment was to partially disrupt the pomace cell wall structure, thereby facilitating subsequent alkaline extraction of both film-forming polysaccharides and bioactive compounds, with the ultimate goal of obtaining films with enhanced antioxidant properties. Accordingly, the selection of treatments was based on achieving a balance between cell wall disruption and the retention of potentially useful components in the solid fraction intended for alkaline extraction. Hemicellulase exhibited the lowest reducing sugar release among the individual enzymes, indicating relatively limited hydrolytic effectiveness under the applied conditions. Although pectinase showed the highest reducing sugar release among the individual enzymes, it also resulted in the lowest soluble dietary fiber content in the remaining solid fraction (3.14%). This may suggest partial hydrolysis and transfer of pectin components into the liquid fraction, which was removed before alkaline extraction. Therefore, cellulase was selected as the individual enzymatic pretreatment, while MIX was selected because it combined the highest reducing sugar release with relatively high soluble dietary fiber content in the remaining solid fraction (11.30%), suggesting extensive enzymatic hydrolysis without a corresponding pronounced reduction in the retained soluble dietary fiber fraction. Thus, CEL and MIX were selected to represent two different enzymatic pretreatment strategies: targeted modification of the cell wall structure using cellulase alone and combined enzymatic modification involving the simultaneous action of enzymes targeting different cell wall components.

**Figure 3:**
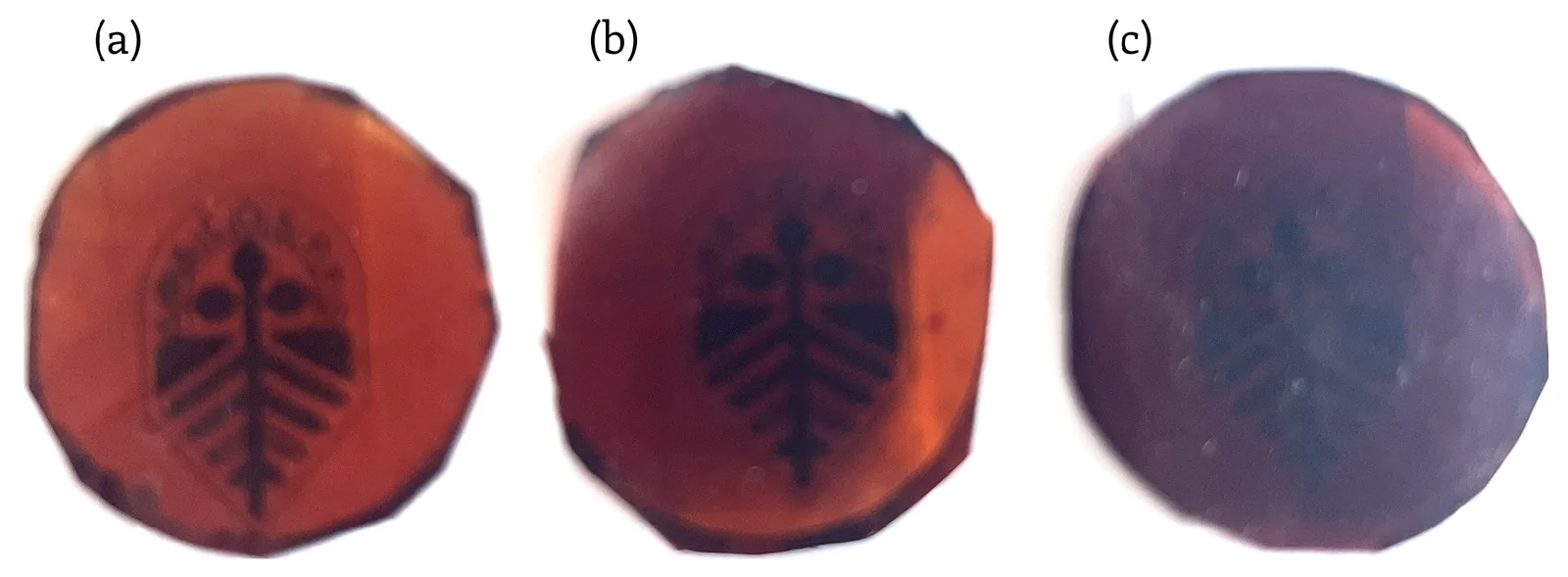
Appearance characteristics of film: (**a**) UNTREATED-film, (**b**) CEL-film, (**c**) MIX-film.

The films obtained from cherry pomace without enzymatic pretreatment, cherry pomace treated with cellulase, and cherry pomace treated with the enzyme mixture were designated as UNTREATED-film, CEL-film, and MIX-film, respectively. Films were prepared from a suspension containing pomace in sodium hydroxide solution, with the aim of achieving alkaline de-esterification of pectin, partial dissolution of hemicellulose, and solubilization of proteins, phenolic compounds, cutin, and lipids, to form an active composite biopolymer film with improved barrier properties against water vapor and antioxidant activities. The appearance of the film samples is shown in Figure 3. The UNTREATED-film was the most transparent and appeared slightly elastic. The CEL-film was slightly darker than the UNTREATED-film and was more brittle and less flexible. The MIX-film was the darkest and appeared firm.

### 2.7. FTIR Spectra of Film Obtained from Untreated and Enzyme-Treated Sour Cherry Pomace

**Figure 4:**
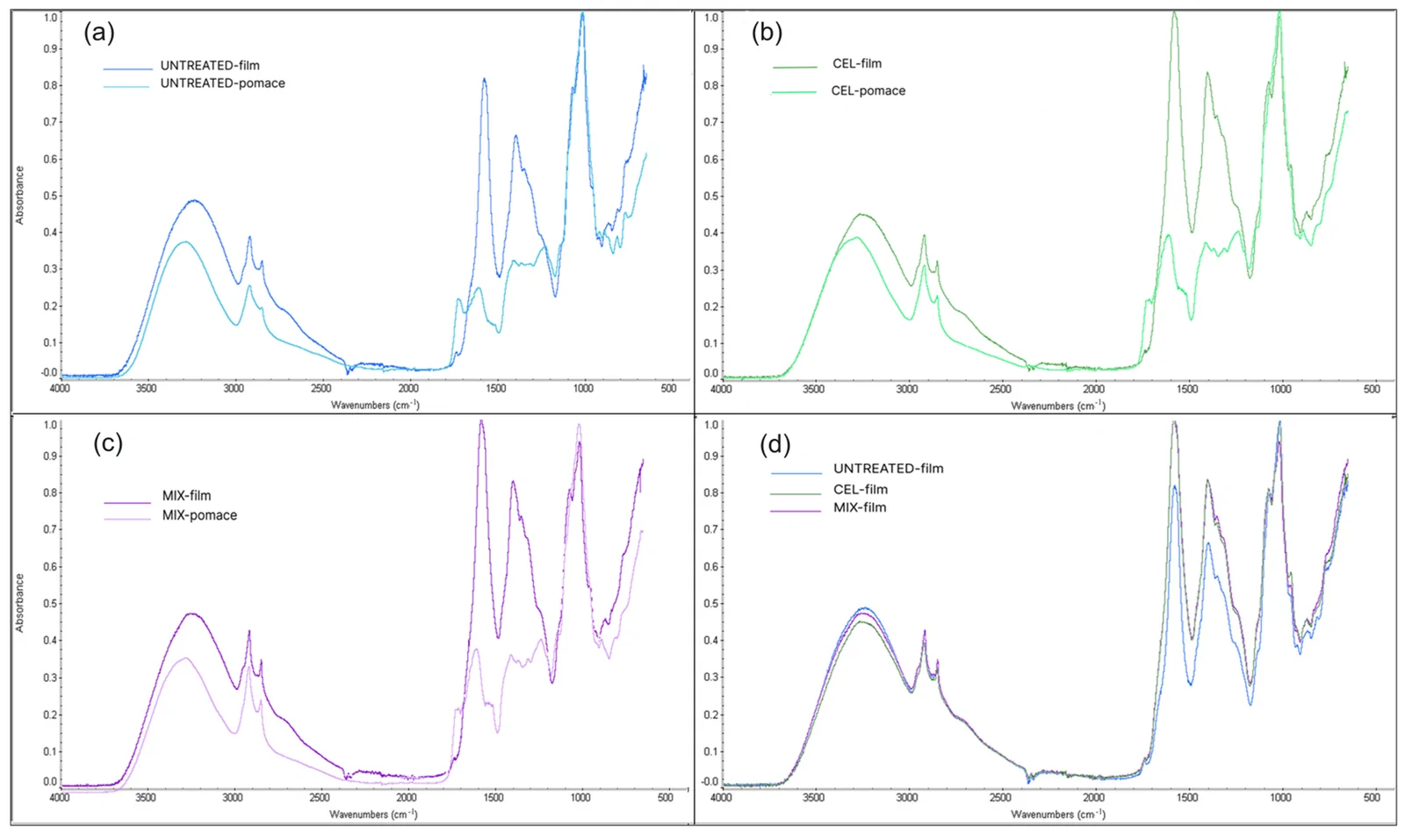
FTIR spectra of pomaces and films: (**a**) untreated film and pomace; (**b**) cellulase-treated film and pomace; (**c**) enzyme mixture-treated film and pomace; (**d**) untreated, cellulase-treated and enzyme mixture-treated films.

In Figure 4a–c, a common change is observed in all three pomace–film pairs. The films exhibit more pronounced bands around ~1600 cm^−1^ and ~1410–1430 cm^−1^, commonly associated with asymmetric and symmetric stretching vibrations of carboxylate (COO^−^) groups, respectively. Together with the reduced contribution of the ester carbonyl band around ~1730 cm^−1^, these spectral changes are consistent with an increased relative contribution of de-esterified pectin structures in the films, which is expected under the alkaline extraction conditions applied in this study. However, these bands may also include contributions from other components present in the complex film matrix, such as proteins, hemicelluloses and other carboxylate-containing compounds, whose characteristic vibrations may overlap with those attributed to de-esterified pectin structures.

Compared to the corresponding pomaces, the peak at about 3300 cm^−1^ is significantly higher and broader in the films. This band is associated with O–H stretching vibrations and may reflect differences in hydrogen bonding interactions and/or moisture content. Aliphatic C–H bands (~2920 and ~2850 cm^−1^) remain present in the films and may originate from the polysaccharide skeleton, with a possible contribution from residual aliphatic components (waxes, cutin). Bands around ~1231 cm^−1^, as well as in the ~818 and ~772 cm^−1^ regions, may include contributions of aromatic structures that may originate from polyphenols and/or soluble lignin fragments. In contrast, the characteristic lignin-associated band around ~1510 cm^−1^ is not clearly expressed in the films, suggesting a relatively low contribution of lignin-derived aromatic structures to the extracted film-forming solutions and the resulting dry films. Overall, the spectra indicate that the films are rich in polysaccharide structures containing carboxylate groups, consistent with the presence of de-esterified pectic components, together with possible contributions from hemicelluloses and minor aromatic and aliphatic constituents. Characteristic protein peaks are covered and masked by polysaccharide peaks, so it can only be assumed that proteins are present in the film due to the extraction conditions, which favors protein extraction.

A comparison of the spectra of UNTREATED, CEL and MIX-films shows a very similar pattern, without the appearance of new bands or the disappearance of existing ones, indicating that all films have a similar basic chemical composition. The differences mainly concern the intensity and shape of individual peaks. It is particularly noticeable that the films obtained after enzymatic treatments (Figure 4d, CEL-film and MIX-film) show higher intensity of the bands around ~1651, ~1615 and ~1410 cm^−1^ compared to the UNTREATED-film. These bands correspond to vibrations of carboxylate groups (COO^−^), indicating a greater contribution of carboxylate-containing structures, including de-esterified pectin components, in the enzymatically treated samples. In addition, the entire region 1200–1800 cm^−1^ shows increased intensity in CEL and MIX-films compared to the UNTREATED-film, which may indicate a higher relative contribution of carboxylate and aromatic components. One possible explanation is that enzymatic disruption of the pomace cell wall matrix facilitated subsequent alkaline extraction of these components.

### 2.8. Characterization of Film Obtained from Untreated and Enzyme-Treated Sour Cherry Pomace

Characterization of film obtained from untreated and enzyme-treated sour cherry pomace was performed (film thickness, water contact angle, water vapor permeability), and the results are presented in Table 4. Film thickness varied from 0.079 to 0.083 mm, but no statistically significant differences were found in the obtained values. The water contact angle of the tested films was 47.32° ± 4.47° for the UNTREATED-film, 50.97° ± 11.50° for the CEL-film, and 44.97° ± 1.94° for the MIX-film. All measured contact angles were well below 90°, indicating hydrophilic surface properties. Although slight differences in mean values were observed, they were not statistically significant (*p* > 0.05). The obtained values were somewhat lower than those typically reported for pectin films (55–65°) [15,29,30,31]. This may be attributed to alkaline de-esterification of pectin, which increases the number of free COO− groups, resulting in a lower water contact angle or higher hydrophilicity of the films. Water vapor permeability (WVP) of the films was (1.69 ± 0.028) × 10^−10^ g/m·s·Pa for the UNTREATED-film, (1.42 ± 0.102) × 10^−10^ g/m·s·Pa for the CEL-film, and (1.49 ± 0.012) × 10^−10^ g/m·s·Pa for the MIX-film. A decrease in WVP was observed in films derived from enzyme-treated pomace, with those derived from untreated pomace. This trend may be associated with structural differences identified by FTIR analysis, indicating variations in component distribution among the films. The obtained WVP values are comparable to those reported for pectin films under similar test conditions (1.3 × 10^−10^ g/m·s·Pa to 5.56 × 10^−10^ g/m·s·Pa) [32,33,34,35]. Given that the wetting angle indicated greater hydrophilicity of the surfaces of all three tested films compared to pectin films, it is possible that the WVP values, which is close to the lower end of the permeability range for pectin films, results from the composite structure of the film.

The contents of polyphenolic and anthocyanin compounds in the obtained films were determined, and the results are presented in Table 4. The highest values were observed in the MIX-film, while both CEL and MIX-films showed higher contents compared to the UNTREATED-film. These differences can be attributed to the applied enzymatic treatment prior to alkaline extraction. Enzymes likely facilitated the breakdown of the cell wall structure, making bound phenolic compounds more accessible and easier to extract. In this context, it can be assumed that the polyphenolic acids identified in the initial extract represent only a fraction of the total polyphenolic content in sour cherry pomace, while a considerable amount remains bound within the polysaccharide matrix, which is consistent with the literature [36]. Additionally, the combination of enzymes had a particularly strong effect on the extraction efficiency of these compounds, which can be explained by the combined activity of different enzymes that more effectively degrade complex cell wall polysaccharides, enhancing solvent accessibility and the release of bound phenolics and anthocyanins. This observation aligns with literature findings demonstrating that different enzymes vary in their ability to release individual phenolic acids, highlighting the enzyme-specific nature of phenolic extraction [37,38] and the potential of enzymatic treatment to significantly improve the recovery of phenolic compounds from plant-based materials. Overall, the results suggest that enzymatic treatment enhances the release of bioactive compounds by improving cell wall degradation and extraction efficiency, while the subsequent alkaline step further supports the release of bound forms through the cleavage of ester linkages.

The antioxidant properties of the obtained films were assessed through ABTS radical scavenging and metal chelating activities. The results were compared and are summarized in Table 4. The ABTS radical scavenging activities of the UNTREATED-film, CEL-film, and MIX-film were 57.36%, 75.79%, and 86.84%, corresponding to 11.27, 14.88, and 17.06 mg TE/g, respectively. As shown, the CEL-film and MIX-film showed statistically significantly higher antioxidant activity compared to the UNTREATED-film, which showed the lowest antioxidant activity, while the MIX-film exhibited the highest antioxidant activity in both in vitro antioxidant tests. These activities were higher than the maximum antioxidant activity previously obtained for bioactive chitosan–polysaccharide composite films by the same method [39]. These results may correlate with the content of polyphenolic and anthocyanin compounds in these films, as the highest antioxidant activity was observed in the MIX-film, which also had the highest identified content of polyphenolic and anthocyanin compounds. In addition, the ABTS radical scavenging activity of the pectin-based films developed in this study, expressed as milligrams of Trolox equivalents per gram of dried film (mg TE/g), was comparable to that reported for pectin films containing individually incorporated phenolic acids [40]. The ABTS activity of the MIX-film (17.06 mg TE/g) was similar to that of films containing gallic acid (17.38 mg TE/g), while the CEL-film (14.8 mg TE/g) exhibited an intermediate value between those reported for films containing caffeic acid and gallic acid. Moreover, all films developed in this study exhibited higher ABTS activity than the control pectin film (0.36 ± 0.12 mg TE/g) reported by Mikus and Galus [40]. These findings highlight the potential of sour cherry pomace-derived phenolic compounds to impart antioxidant properties to pectin-based films.

The chelating ability of the obtained films was evaluated, as the capacity to bind transition metals is important for reducing oxidation in food systems [41]. The results indicated that all films were capable of chelating iron (II), with a maximum ability of 89.67% measured for the MIX-film. This activity was higher than the maximum chelating ability reported for chitosan film enriched with essential oils [41]. Furthermore, the phenolic compounds present in the films are closely associated with their metal chelating activity [40], but the intense activities observed for all examined films can also be attributed to the presence of de-esterified pectin forms identified by FTIR analysis, which is consistent with the literature [42]. The antioxidant properties demonstrated by all obtained films indicate their potential for food preservation applications [43].

### 2.9. pH Responsiveness Potential

As demonstrated by the results (Table 5), sour cherry pomace represents a valuable source of anthocyanins, which were detected in all prepared films. To further evaluate their functional potential, the films were exposed to three different pH conditions to assess their ability to change color in response to environmental variations. The color difference (ΔE) was calculated for each film at each pH value relative to the initial film. The results revealed noticeable differences in visual appearance among the films. The MIX-film exhibited the darkest color, while the UNTREATED-film was the lightest, with the CEL-film showing intermediate behavior. The lower L* values observed for the MIX and CEL-films compared with the UNTREATED-film are consistent with their higher content of anthocyanins and other phenolic compounds. These color differences were mainly associated with the presence of natural chromophore compounds, as also reported in previous studies [15]. In addition to phenolic compounds, anthocyanins are known to interact with the cell wall, which can result in the entrapment of these compounds within the polysaccharide matrix, reducing their extractability [44]. In this context, the application of hydrolytic enzymes likely promotes partial degradation and restructuring of the cell wall, improving the release and availability of anthocyanins. This can also explain the observed differences in color intensity and pH responsiveness between the MIX and UNTREATED-films. Furthermore, due to structural transformations of the anthocyanins present and their color changes under different pH conditions, variation in ΔE occurred, and the obtained values indicated a slightly lower change compared to films with added chromogenic agents [45]. Specifically, maximal pH sensitivity was observed at pH 5 for the MIX-film (ΔE = 41.79), which was almost within the range of films based on soy protein isolate with grape skin red at the same pH [45]. These results provide a solid basis for developing pH-sensitive packaging systems from sour cherry pomace without adding chromophores.

## 3. Materials and Methods

### 3.1. Chemicals

n-Hexane (VWR Chemicals, Rosny-sous-Bois, France); cc H_2_SO_4_ (Merck, Darmstadt, Germany); NaOH (Centrohem, Stara Pazova, Serbia); HCl (Merck, Darmstadt, Germany); NaOCH_3_ (Thermo Fisher, Waltham, MA, USA); anhydrous methanol (Thermo Fisher, Waltham, MA, USA); chloroform (VWR Chemicals, Leuven, Belgium); anhydrous Na_2_SO_4_ (Centrohem, Stara Pazova, Serbia); nitrogen (Messer Tehnogas, Belgrade, Serbia); anhydrous pyridine (Alfa Aesar, Ward Hill, MA, USA); BSTFA containing 1% TMCS (Alfa Aesar, Ward Hill, MA, USA); CuSO4·5H_2_O (Centrohem, Stara Pazova, Serbia); K_2_SO_4_ (Merck, Darmstadt, Germany); sodium bromide (Centrohem, Stara Pazova, Serbia); sodium acetate (Centrohem, Stara Pazova, Serbia); acetic acid (Centrohem, Stara Pazova, Serbia); trolox (Merck KGaA, Darmstadt, Germany); Cellulase from *Aspergillus niger* (product no. 22178; declared activity ~0.8 U/mg); pectinase from *Aspergillus sp.* (product no. 89216; declared activity ≥0.3 U/mg); and hemicellulase from *Aspergillus niger* (product no. H2125; activity 1.5 U/mg) were purchased from Sigma-Aldrich (Merck KGaA, Darmstadt, Germany). All reagents were of analytical grade.

### 3.2. Materials

The sour cherry pomace used as raw material in this study was kindly supplied by the local food and beverage manufacturer Nectar d.o.o. (Bačka Palanka, Serbia). The enzyme used in the treatments of sour cherry pomace were as follows: cellulase (*Aspergillus niger*, Sigma), pectinase (*Aspergillus* sp., Sigma) and hemicellulase (*Aspergillus niger*, Sigma).

### 3.3. Enzymatic Treatments of Sour Cherry Pomace

Enzymatic treatment of sour cherry pomace was performed using commercial cellulase, pectinase, and hemicellulase, applied individually or in combination (MIX). For each treatment, dried sour cherry pomace was suspended in 0.1 mol/L acetate buffer (pH 5), prepared from sodium acetate and acetic acid, using a solid-to-liquid ratio of 1:10 (*w*/*v*), calculated on the mass of dry pomace relative to the volume of buffer. The resulting suspensions were incubated at 50 °C for 1 h under continuous stirring at 1000 rpm using an IKA RTC Standard (IKA-Werke GmbH & Co. KG, Staufen, Germany) magnetic stirrer. Control samples were subjected to the same conditions in the absence of enzymes, with acetate buffer alone. Each enzyme was applied individually at 1% (*w*/*w*), based on the dry weight of the sour cherry pomace. For the MIX treatment, the three enzymes (cellulase, pectinase, and hemicellulase) were combined in equal proportions, each at a concentration of 0.333% (*w*/*w*), resulting in a total enzyme concentration of 1% (*w*/*w*), based on the dry weight of the sour cherry pomace. Following incubation, the suspensions were filtered under vacuum to separate the liquid and solid fractions. The solid residues were subsequently dried in an oven at 60 °C, whereas the liquid fractions were frozen and stored until further analyses. The concentration of reducing sugars released into the liquid fraction was determined using the 3,5-dinitrosalicylic acid (DNS) method [46], with glucose as the standard. The recovered solid fractions were used for other analysis and, in the case of the selected treatments (CEL and MIX), subsequent alkaline extraction and film preparation.

### 3.4. Proximate Analysis of Sour Cherry Pomace

#### 3.4.1. Moisture and Ash

Moisture content was determined by oven drying to constant weight at 103 ± 2 °C, using a procedure adapted from previously reported methods [47,48]. Approximately 10.00 g of sample was placed in pre-dried, pre-weighed glass weighing bottles with lids. During drying, lids were kept slightly open to allow moisture release. Samples were dried to constant mass, cooled in a desiccator with lids closed, and reweighed. Moisture content was calculated from the mass loss and expressed on a wet basis (g/100 g sample), according to the following equation:

$$M\;\left(\%\right)=\frac{m_{i}-m_{d}}{m_{i}}\cdot 100$$

where *M* is the moisture content (%), *m_i_* is the initial sample mass (g) and *m_d_* is the sample mass after drying (g). Ash content was determined by dry ashing, using a procedure adapted from previously reported methods [47,48]. About 1.00 g of sample was weighed into pre-weighed crucibles, gently charred over a low flame, and incinerated in a muffle furnace at 550 °C for approximately 4 h, until white ash of constant mass was obtained. Crucibles were cooled in a desiccator and weighed. Ash content was calculated as the mass of inorganic residue and expressed on a dry weight basis (g/100 g DW), according to the following equation:

$$A\;\left(\%\right)=\frac{m_{a}}{m_{i}(1-\frac{M}{100})}\cdot 100$$

where *A* is the ash content (g/100 g DW), *m_a_* is the mass of ash (g), *m_i_* is the initial sample mass (g), and *M* is the moisture content (%).

#### 3.4.2. Total Lipids

Total lipid content was quantified by Soxhlet extraction, using a procedure adapted from previously reported method [49]. Approximately 5.00 g of dried sample was transferred into a cellulose extraction thimble, and extraction with n-hexane was carried out for 4 h. After extraction, hexane was removed by evaporation, and the lipid residue was dried at 105 °C to constant mass, cooled in a desiccator, and weighed. Results were expressed on a dry weight basis (g/100 g DW), according to the following equation:

$$L\;\left(\%\right)=\frac{m_{l}}{m_{i}(1-\frac{M}{100})}\cdot 100$$

where *L* is total lipid content (g/100 g DW), *m_l_* is the mass of extracted lipids (g), *m_i_* is the initial sample mass (g), and *M* is the moisture content (%).

#### 3.4.3. Proteins

Total protein content was determined by the Kjeldahl method, using a procedure adapted from previously reported methods [50]. Approximately 0.10 g of sample was digested with concentrated H_2_SO_4_ in the presence of a CuSO_4_/K_2_SO_4_ catalyst mixture until a clear digest was formed. After cooling, the digest mixture was alkalized with 33% NaOH to liberate ammonia (NH_3_), which was distilled and collected in 0.01 mol/L HCl. Excess acid was back-titrated with standardized 0.01 mol/L NaOH. Total nitrogen content was calculated and converted to protein content using a factor of 6.25:

$$P\;\left(\%\right)=6.25\cdot \frac{(V_{b}-V_{s})\cdot c\cdot 1.4007}{m_{i}(1-\frac{M}{100})}$$

where *P* is protein content (g/100 g DW), *V_b_* and *V_s_* are the volumes of NaOH (mL) consumed for the blank and sample, respectively, *c* is the NaOH concentration (mol/L), *m_i_* is the initial sample mass (g), and *M* is the moisture content (%). Results are expressed on a dry weight basis (g/100 g DW).

#### 3.4.4. Carbohydrates

Total carbohydrates were calculated by subtracting the determined contents of proteins, lipids, and ash from the dry matter, using the mean values of protein, lipids and ash contents, all expressed on a dry weight basis (g/100 DW):

*C* (%) = 100 − (*P* + *L* + *A*)

where *C* is the total carbohydrate content, and *P*, *L*, and *A* represent protein, total lipid, and ash contents, respectively, all expressed in g/100 g DW.

### 3.5. Analytical Methods

#### 3.5.1. Dietary Fiber

Insoluble (IDF), and soluble dietary fiber (SDF) contents were determined using the enzymatic–gravimetric method (AACC 32-05.1) [51]. First, the samples were treated with digestive enzymes to remove starch and proteins; after that, IDF is collected by filtration, washed, dried, and weighed. SDF is determined from the filtrate by precipitation with ethanol, followed by filtration, drying, and weighing and the results were expressed as percentages on a dry matter basis of the initial sour cherry pomace.

#### 3.5.2. Total Polyphenolic and Anthocyanin Contents

The extraction of polyphenolic substances from dried cherry pomace was performed using 50% methanol aqueous solution. Sample of cherry pomace (500 mg) was extracted in two steps: with 10 mL mixing for 5 min on vortex (Heldolph, Schwabach, Germany) and for 30 min in an ultrasound bath (Elmasonic, Singen, Germany). The extractions of polyphenolic substances from UNTREATED-film, CEL-film, and MIX-film were performed using a phosphate buffer solution (0.1 mol/L, pH 7) for 24 h with a solid-to-liquid ratio of 1:100 (*w*/*v*). The suspension was centrifuged at 5000 rpm for 5 min, and the supernatant was subjected to further analysis.

#### 3.5.3. Determination of Bioactive Compound Content

Samples were subjected to spectrophotometric assays to determine their total polyphenolic (TPC) and anthocyanin (TAC) contents. The Folin–Ciocalteu method was used for TPC determination [52], while TAC was determined according to Lee et al. [53]. Total polyphenolic content in the samples was determined spectrophotometrically using the Folin–Ciocalteu (FC) method adapted to the microscale [54]. Briefly, the reaction mixture was prepared in a 96-well microplate by mixing 15 μL of the sample, 170 μL of distilled water, 12 μL of Folin–Ciocalteu reagent (2 mol/L), and 30 μL of 20% sodium carbonate. After 1 h, room temperature, dark conditions, and the absorbance at 750 nm were measured using distilled water as a blank. The results are expressed as mg gallic acid equivalents (GAE) per g of dried plant material (mg GAE/g DW). The total anthocyanin contents in extracts were estimated spectrophotometrically using the pH differential method adapted to the microscale [54]. For TAC, absorbances were measured at a wavelength of 510 and 700 nm, alongside their blanks. Anthocyanins were expressed in terms of cyanidin-3-glucoside equivalents per g of dry material (mg CyGE/g DW).

#### 3.5.4. HPLC DAD Analysis

Dried cherry pomace extract was subjected to high-performance liquid chromatography (HPLC) analysis coupled with diode array (DAD) detector (Shimadzu Prominence, Kyoto, Japan), to identify and quantify the polyphenolic compounds [55]. Prior to analysis, prepared extract was filtered through a 0.45 μm membrane filter. Mobile phase A consisted of acetonitrile and mobile phase B of 1% formic acid. The flow rate was 1 mL/min, and the gradient program was as follows: 10–25% A from 0 to 10 min, 25–60% A from 10 to 20 min, and 60–70% A from 20 to 30 min, followed by a return to the initial conditions over 10 min and equilibration for 5 min. The separation was carried out on a Luna C-18 RP column (5 μm, 250 × 4.6 mm, Phenomenex, Torrance, CA, USA), fitted with a C18 guard column (4 × 30 mm) from the same supplier. Different wavelengths for specific compounds were used to record chromatograms (280 nm for hydroxybenzoic acids, 320 nm for hydroxycinnamic acids and 360 nm for flavonoids) and all results were calculated as mg per g of dried plant material. Compounds were identified and quantified using reference standards prepared in 50% methanol and filtered through a 0.45 μm membrane filter. Polyphenolic compounds (gallic acid, caffeic acid, coumaric acid, sinapic acid, rutin, and ellagic acid) were identified by matching the retention time and their spectral characteristics against those of standards. The external standard method was the technique used for quantification. For each compound, a stock solution was made by accurately weighing out commercial standard of polyphenolic compounds followed by dissolution in 50% methanol. Five different concentrations of standard solutions from a concentration 1 mg/mL were prepared to make calibration range for each phenolic compound. Different aliquots of standard solutions were transferred into vials and subjected to HPLC analysis in order to generate calibration curves. Peak areas from chromatograms were plotted against known concentrations of standards. Equations generated via linear regression were used to establish concentrations of polyphenolic compounds in samples. All analyses were run in triplicate and data acquisition was carried out by the LC Solution Software version 1.25 (Shimadzu, Kyoto, Japan).

### 3.6. Fourier-Transform Infrared Spectroscopy (FTIR)

The structural properties dried pomace and films were determined using Nicolet IS10 FT-IR spectrophotometer (Thermo Fisher Scientific, Waltham, MA, USA) using attenuation total reflection (ATR) technique. Analyzed spectra were recorded in the spectral range 4000–400 cm^−1^ at a resolution of 4 cm^−1^ taking 16 scans per record. Before scanning sample spectra, background spectra was collected each time. Omnic 8.1. (Thermo Fisher Scientific, Waltham, MA, USA) software was used to process collected FTIR spectra.

### 3.7. Film Formation

Three starting pomaces were used: untreated sour cherry pomace, cherry pomace treated with cellulase, and cherry pomace treated with the enzyme mixture. Each was used to prepare a filmogenic suspension containing 2.5% pomace in 0.1 mol/L NaOH. The base extraction of the cherry pomaces was carried out for 24 h at room temperature. After extraction, the film-forming suspension was filtered, and the filtrate was used as the film-forming solution. The film-forming solution was cast onto Petri plates lined with Teflon cloth at a mass-to-surface ratio of 0.15 g/cm^2^, after which the films were left on a leveled flat surface for 48 h to dry under room conditions (23 ± 2 °C and RH 50 ± 5%). The dry films were then peeled off and stored in a refrigerator until testing. Before testing, the films were conditioned for at least 48 h at 23 ± 2 °C and RH 50 ± 5%.

### 3.8. Characterization of Film Physical Properties

#### 3.8.1. Film Thickness

Film thickness was measured using a micrometer Digico 1, with sensitivity of 0.001 mm (Tesa, Renens, Switzerland), as described in ASTM D6988-21 [56].

#### 3.8.2. Water Contact Angle

The surface energy measurement of obtained films was performed using the water contact angle measurement in ImageJ software 1.54g with the DropSnake plugin, using a method described and validated by Senturk Parreidt et al. [57].

#### 3.8.3. Water Vapor Barrier Properties

Water vapor barrier properties of films were determined gravimetrically (dish method), according to the ISO 2528:1995 [58]. The testing conditions were as follows: temperature 23 ± 2 °C and relative humidity 57 ± 1% using sodium bromide saturated solution in the cup and dry silica gel in the outer atmosphere, (0% RH).

### 3.9. Antioxidant Activities of Film

The extraction of bioactive components from dried films was performed using a phosphate buffer solution (0.1 mol/L, pH 7) for 24 h. The suspension was centrifuged at 5000 rpm for 5 min, and the supernatant was subjected to further analysis. The antioxidant activities of the obtained films were evaluated using ABTS radical scavenging activity in vitro assays [59] at a sample concentration of 5 mg/mL and metal chelating activity [60] at a sample concentration of 0.5 mg/mL. The obtained results in both tests are expressed as percentages. For better comparison with previously published studies, the ABTS results are also expressed as mg TE/g, based on the Trolox calibration curve, which showed a slope of 0.2545%·L/µmol and a coefficient of determination (R^2^) of 0.999.

### 3.10. pH Responsiveness Potential

As the obtained film contained naturally occurring pigments, its pH-responsive colorimetric behavior was evaluated in solutions at different pH values following the approach described by Hailu et al. [61] with modification. At room temperature, to test the color of the indicator film sample, 0.1 mL of each pH buffer solution (pH 3.5, pH 5 and pH 7) was dropped on approximately 1 cm^2^ of film and dried at room temperature. The different color changes of the dried film were measured by Minolta Chromameter (CR-400, Minolta Co., Osaka, Japan).

### 3.11. Statistical Analysis

All tests were performed in three independent replicates. Statistical analysis data were presented as means with the standard deviation (SD). One-way analysis of variance followed by the Tukey test was used to assess differences among the means of different samples and was performed in Statistica software, version 14. Results with *p* < 0.05 are considered statistically different and are denoted by different letters. Formal tests of normality and homogeneity of variances were not performed due to the limited number of replicates (*n* = 3 per group).

## 4. Conclusions

Sour cherry pomace represents a promising and sustainable source of biopolymers that can be used to produce bioactive and pH-responsive films due to its innate structural components, such as polysaccharides, antioxidants, and pigments. In this study, enzymatic treatment with hydrolytic enzymes gently modified sour cherry pomace, improving the availability of endogenous bioactive compounds, including phenolics and anthocyanins, during subsequent extraction. Among the investigated treatments, the combined enzymatic treatment proved particularly effective in enhancing the recovery of endogenous phenolic compounds and anthocyanins, yielding films with improved antioxidant activity and demonstrating the potential of sour cherry pomace as a multifunctional, naturally bioactive film-forming material with pH-responsive properties. The simultaneous production of active and pH-responsive films using naturally occurring pigments and phenolic compounds avoids compatibility issues associated with incorporating separate bioactive and pigment additives, while potentially minimizing adverse effects on film structural integrity and functional performance. The proposed strategy contributes to the sustainable utilization of agri-food by-products and the development of functional packaging materials. Although the obtained results indicate the potential of the developed films for food packaging applications, their performance was evaluated only under controlled laboratory conditions. Therefore, further studies involving real food systems and storage trials are needed to assess their effectiveness in food preservation. Further studies should also include a comprehensive evaluation of the physical, mechanical, thermal, and barrier properties of the developed films to assess their suitability for practical food packaging applications.

## Figures and Tables

**Table 1:** Untreated sour cherry pomace composition.

Component	Content
Proximate composition	
Moisture (% wb)	10.43 ± 0.19
Ash (% DW)	3.23 ± 0.05
Proteins (% DW)	8.49 ± 0.51
Fats (% DW)	0.93 ± 0.17
Carbohydrates (% DW)	87.35 ± 0.72
Total polyphenolic and anthocyanin contents	
Total polyphenolic content (mg GAE/g DW)	21.25 ± 0.76
Total anthocyanin content (mg Cy3Glu/g DW)	1.28 ± 0.02
Individual polyphenolic contents (mg/100 g DW)	
Gallic acid	5.84 ± 0.05
Caffeic acid	20.61 ± 0.05
Coumaric acid	24.49 ± 0.01
Sinapic acid	36.34 ± 0.06
Rutin	7.73 ± 0.08
Ellagic acid	5.58 ± 0.21

**Table 2:** Proximate composition of buffer and enzyme-treated sour cherry pomace.

Sample	Moisture (% wb)	Ash (% DW)	Proteins (% DW)	Fats (% DW)	Carbohydrates (% DW)
Buffer	3.58 ± 0.05 ^d^	5.62 ± 0.72 ^a^	11.96 ± 1.43 ^a^	1.44 ± 0.10 ^b^	80.98 ± 2.26 ^a^
Cellulase	4.62 ± 0.10 ^c^	6.21 ± 0.11 ^a^	12.25 ± 0.66 ^a^	1.56 ± 0.01 ^b^	79.98 ± 0.77 ^a^
Pectinase	6.46 ± 0.19 ^a^	5.80 ± 0.08 ^a^	12.47 ± 1.06 ^a^	2.01 ± 0.08 ^a^	79.72 ± 1.22 ^a^
Hemicellulase	2.87 ± 0.09 ^e^	5.59 ± 0.53 ^a^	10.95 ± 0.14 ^a^	1.44 ± 0.13 ^b^	82.02 ± 0.80 ^a^
MIX	5.64 ± 0.04 ^b^	6.21 ± 0.05 ^a^	10.67 ± 0.38 ^a^	1.97 ± 0.07 ^a^	81.15 ± 0.40 ^a^

Different letters in the table represent the significant differences according to Tukey’s HSD test (*p* < 0.05).

**Table 3:** Dietary fiber content in buffer and enzyme-treated sour cherry pomace.

Sample	IDF (%)	SDF (%)
Buffer	22.86 ± 0.38 ^a^	8.82 ± 1.23 ^ab^
Cellulase	17.29 ± 0.61 ^c^	7.15 ± 0.61 ^bc^
Pectinase	21.12 ± 0.13 ^ab^	3.14 ± 0.17 ^c^
Hemicellulase	19.78 ± 0.88 ^bc^	12.46 ± 0.12 ^a^
MIX	19.71 ± 0.39 ^bc^	11.30 ± 1.02 ^ab^

Different letters in the table represent the significant differences according to Tukey’s HSD test (*p* < 0.05).

**Table 4:** Comparative analysis of physical, barrier and bioactive properties of films.

Parameter	UNTREATED-Film	CEL-Film	MIX-Film
Thickness (mm)	0.083 ± 0.003 ^a^	0.079 ± 0.004 ^a^	0.082 ± 0.003 ^a^
Total polyphenolic content (mg GAE/g DW)	18.21 ± 0.26 ^c^	22.10 ± 0.39 ^b^	28.98 ± 0.21 ^a^
Total anthocyanin content (mg Cy3Glu/g DW)	0.36 ± 0.01 ^c^	1.27 ± 0.01 ^b^	1.52 ± 0.01 ^a^
Water contact angle (°)	47.32 ± 4.47 ^a^	50.97 ± 11.50 ^a^	44.97 ± 1.94 ^a^
Water vapor permeability (g/m·s·Pa)	(1.69 ± 0.028) × 10^−10 a^	(1.42 ± 0.102) × 10^−10 b^	(1.49 ± 0.012) × 10^−10 b^
ABTS activity (%)	57.36 ± 4.74 ^b^	75.79 ± 2.10 ^a^	86.84 ± 0.53 ^a^
Chelating activity (%)	75.00 ± 1.08 ^b^	85.87 ± 1.08 ^a^	89.67 ± 1.63 ^a^

Different letters in the table represent the significant differences according to Tukey’s HSD test (*p* < 0.05).

**Table 5:** pH responsiveness potential of film.

Sample	L*	a*	b*	ΔE	Photo
UNTREATED-film	39.51 ± 0.33 ^d^	27.99 ± 0.24 ^a^	18.99 ± 0.31 ^c^	-	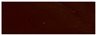
pH 7	49.38 ± 0.01 ^c^	27.98 ± 0.09 ^a^	38.77 ± 0.13 ^b^	22.10 ± 0.02 ^c^	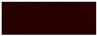
pH 5	56.05 ± 0.20 ^b^	24.51 ± 0.14 ^b^	46.35 ± 0.01 ^a^	32.16 ± 0.19 ^b^	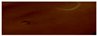
pH 3.5	58.08 ± 0.21 ^a^	20.65 ± 0.26 ^c^	46.13 ± 0.20 ^a^	33.69 ± 0.24 ^a^	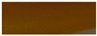
CEL-film	33.72 ± 0.04 ^c^	17.60 ± 0.32 ^b^	5.86 ± 0.25 ^d^	-	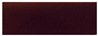
pH 7	47.86 ± 1.03 ^a^	26.74 ± 0.98 ^a^	33.94 ± 1.35 ^a^	32.77 ± 0.66 ^a^	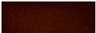
pH 5	40.13 ± 0.14 ^b^	30.32 ± 0.27 ^a^	23.90 ± 0.44 ^b^	22.99 ± 0.84 ^b^	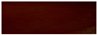
pH 3.5	47.73 ± 0.21 ^a^	20.83 ± 1.57 ^b^	14.89 ± 1.95 ^c^	17.17 ± 1.38 ^b^	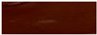
MIX-film	30.85 ± 0.86 ^c^	14.96 ± 0.16 ^c^	3.6 ± 0.28 ^c^	-	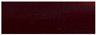
pH 7	41.81 ± 0.09 ^b^	30.11 ± 0.27 ^a,b^	29.29 ± 0.19 ^b^	31.80 ± 0.25 ^b^	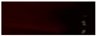
pH 5	48.73 ± 0.46 ^a^	29.04 ± 0.29 ^b^	38.63 ± 0.62 ^a^	41.79 ± 0.72 ^a^	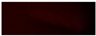
pH 3.5	41.22 ± 0.28 ^b^	30.60 ± 0.32 ^a^	31.27 ± 0.93 ^b^	33.45 ± 1.05 ^b^	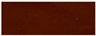

Different letters in the table represent the significant differences according to Tukey’s HSD test (*p* < 0.05). ΔE = ((△L*)^2^ + (△a*)^2^ + (△b*)^2^)^1/2^, △L*: brightness difference, △a*, △b*: colorimetric difference.

## Data Availability

The original contributions presented in this study are included in the article. Further inquiries can be directed to the corresponding author.
